# Inosine pranobex enhances human NK cell cytotoxicity by inducing metabolic activation and NKG2D ligand expression

**DOI:** 10.1002/eji.201847948

**Published:** 2019-09-12

**Authors:** Michael T. McCarthy, Da Lin, Tomoyoshi Soga, Julie Adam, Christopher A. O'Callaghan

**Affiliations:** ^1^ Wellcome Trust Centre for Human Genetics, Nuffield Department of Medicine University of Oxford Oxford OX3 7BN UK; ^2^ Institute for Advanced Biosciences Keio University Tsuruoka Yamagata 997‐0052 Japan; ^3^ Target Discovery Institute Nuffield Department of Medicine University of Oxford Oxford OX3 7FZ UK

**Keywords:** immunomodulation, innate immunity, inosine pranobex, MICA, NKG2D

## Abstract

Inosine pranobex (IP) is a synthetic immunomodulating compound, indicated for use in the treatment of human papillomavirus‐associated warts and subacute sclerosing panencephalitis. Previous studies demonstrate that the immunomodulatory activity of IP is characterized by enhanced lymphocyte proliferation, cytokine production, and NK cell cytotoxicity. The activation of NKG2D signaling on NK cells, CD8^+^ T cells, and γδ T cells also produces these outcomes. We hypothesized that IP alters cellular immunity through the induction of NKG2D ligand expression on target cells, thereby enhancing immune cell activation through the NKG2D receptor. We tested this hypothesis and show that exposure of target cells to IP leads to increased expression of multiple NKG2D ligands. Using both targeted metabolic interventions and unbiased metabolomic studies, we found that IP causes an increase in intracellular concentration of purine nucleotides and tricarboxylic acid (TCA) cycle intermediates and NKG2D ligand induction. The degree of NKG2D ligand induction was functionally significant, leading to increased NKG2D‐dependent target cell immunogenicity. These findings demonstrate that the immunomodulatory properties of IP are due to metabolic activation with NKG2D ligand induction.

## Graphical abstract

Cellular metabolic activation induces cell surface MICA expression by enhancing purine nucleotide synthesis. We show that inosine pranobex, a licensed antiviral drug, enhances purine nucleotide synthesis, NKG2D ligand expression, and cellular susceptibility to NKG2D‐dependent NK cell cytotoxicity in metabolically active cells.

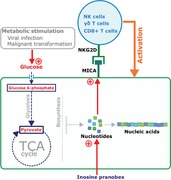

## Introduction

Modulation of the immune response has formed the basis of most recently developed therapies for inflammatory disease, organ transplantation, and strikingly for some cancers. The rational development of new immunotherapies that can be successfully translated into clinical practice is facilitated by a detailed understanding of the underlying molecular immunology.

Inosine pranobex (IP) has been demonstrated to have therapeutic benefit in human papillomavirus‐induced warts 1, measles virus infection resulting in subacute sclerosing panencephalitis (SSPE) 2 and alopecia 3. It is currently licensed for use in the treatment of genital papilloma virus‐induced warts, HSV infections and SSPE in many countries.

Despite this clinical use, the molecular mechanisms underlying IP‐induced immunomodulation remain unclear. Existing studies provide mechanistic insights. IP enhances the proliferative response of lymphocytes exposed to conventional mitogens, including phytohaemagglutinin 4, 5, Con A 6, and anti‐CD3 4, but lacks intrinsic lymphocyte mitogenic capability 7. IP‐treated lymphocytes are both more responsive to, and more productive of, IL‐2 5, 8. While some studies demonstrate IP‐induced enhanced NK cell cytotoxicity 6, other studies have not observed this effect 9. These findings were largely published before the discovery of the NKG2D receptor and its ligands, and do not address the possibility that the effects observed may be mediated through increased triggering of NKG2D by activating ligands on target cells. In most of these studies, the duration of exposure of target cells to IP during the experiments is unclear.

**Figure 1:**
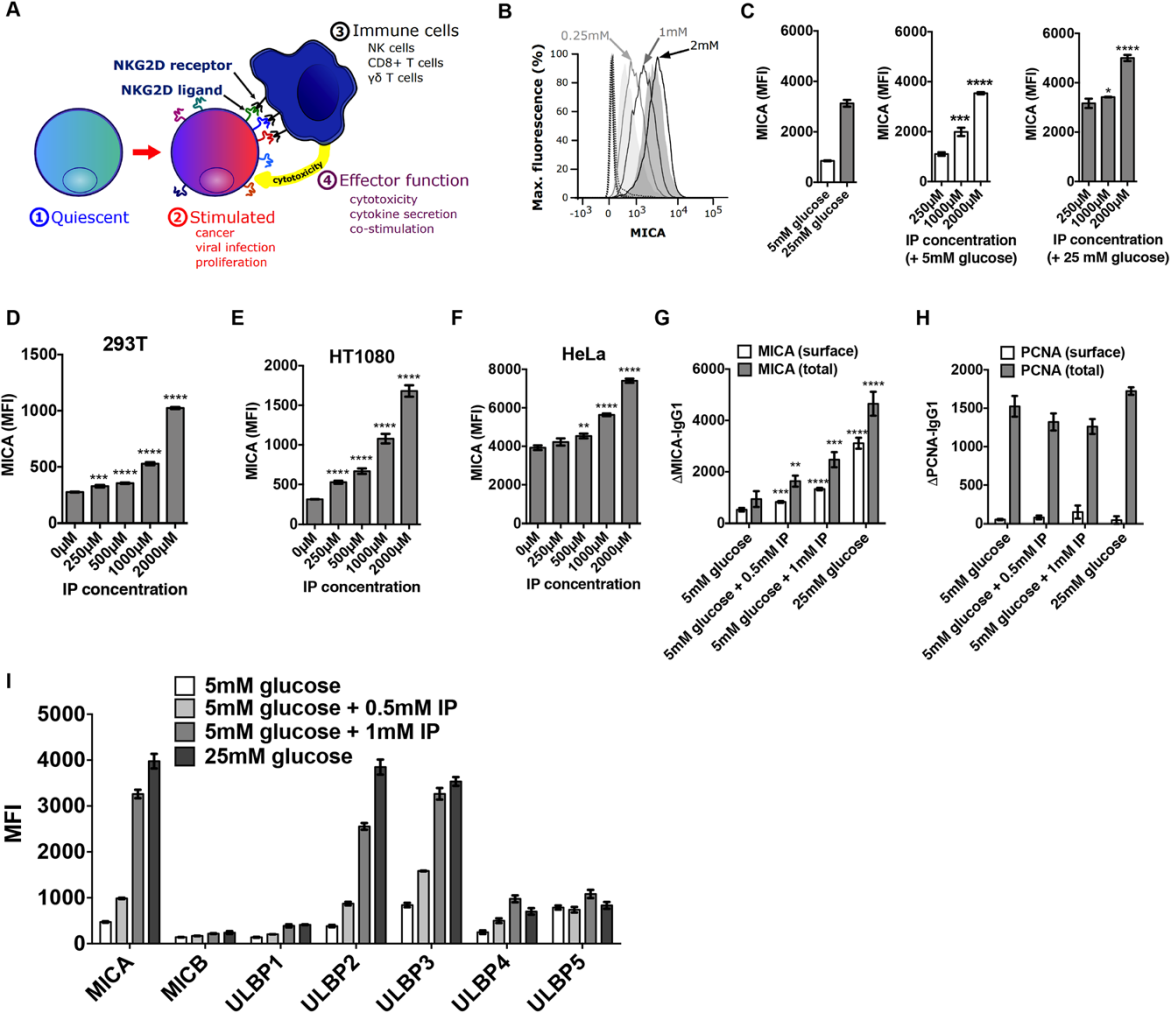
IP induces dose‐dependent cell surface NKG2D ligand expression. (A) NKG2D ligands are not typically expressed on healthy quiescent cells. Stimuli including malignant transformation, viral infection, and proliferative lymphocyte activation are associated with NKG2D ligand induction. Expression can cause cytotoxicity, cytokine secretion, or costimulation through binding to the activating receptor, NKG2D. (B) HEK293T cells were cultured in 5 mM glucose with 0.25, 1, or 2 mM IP for 48 h, and cell surface expression of MICA (2C10) was measured by flow cytometry. A strong dose‐dependent increase in MICA expression was observed. Isotype controls (dotted histogram), cells cultured in 5 mM glucose only (light grey shaded histogram) or in 25 mM glucose (dark grey shaded histogram) are also shown. (C) Cells were cultured in 5 or 25 mM glucose with IP in biological triplicates and MICA expression was measured by flow cytometry. In 5 mM glucose, IP produced a significant increase in cell surface MICA expression compared to untreated cells. In 25 mM glucose, a significant increase in MICA expression was observed at higher IP concentrations. (D) HEK293T cells, (E) HT1080 cells (human fibrosarcoma), and (F) HeLa cells (human cervical carcinoma) demonstrate dose‐dependent MICA (2C10) expression when cultured with IP. (G) We tested whether IP influenced total cellular MICA levels by staining permeabilized and non‐permeabilized cells in parallel. Permeabilized cells displayed the same dose‐dependent IP‐induced MICA expression as non‐permeabilized cells. (H) We tested for adequate cell permeabilization by measuring the expression of PCNA (14‐9910‐80) in both non‐permeabilized and permeabilized cells by flow cytometry. PCNA was only detected in permeabilized cells and did not increase in an IP‐dependent manner. (I) The effect of IP on the induction of multiple NKG2D ligands including MICB (MAB1599), ULBP1 (MAB1380), ULBP2 (MAB1298), ULBP3 (MAB1517), ULBP4 (6E6), and ULBP5 (6D10) was tested by flow cytometry. ^∗^ *p* < 0.05; ^∗∗^ *p* < 0.01; ^∗∗∗^ *p* < 0.001; and ^∗∗∗∗^ *p* < 0.0001. Histograms represent mean and 95% confidence interval. The data shown is from a single experiment that contained three biological replicates. Experiments were performed independently three times with consistent results. Means were compared using *t*‐tests. MFI, mean fluorescence intensity.

NKG2D is an activating receptor expressed predominantly on NK cells, CD8^+^ T cells, γδ T cells, NKT cells, and some CD4^+^ T cell populations 10. A series of eight activating ligands for NKG2D have been identified in humans and these include the MICA, MICB and the RAET1 or ULBP molecules. While the NKG2D ligands are generally not expressed in healthy quiescent cells, expression can be induced in response to several stimuli, including viral infection 11, DNA damage 12, inflammatory cytokines 13, loss of cell adhesion 14, and proliferative cell activation 15. We have previously demonstrated that enhanced purine nucleoside synthesis controls NKG2D ligand upregulation 16. The transcriptional control of NKG2D ligands is incompletely understood, but in the best studied ligand, MICA, it involves intragenic transcriptional interference between tandem promoters 17. Cells expressing NKG2D ligands become targets for immune cells that express the NKG2D receptor 18. Binding between the NKG2D receptor on immune cells and NKG2D ligands on potential target cells can lead to cytotoxicity against these target cells 19, cytokine secretion 20, or co‐stimulation 21 depending on the immune context (Fig. 1A).

We noted an overlap between the reported consequences of IP treatment and the consequences of NKG2D activation, including enhanced lymphocyte proliferation, cytokine secretion, and NK cell cytotoxicity. Therefore, we hypothesized that IP acts by inducing NKG2D ligand expression in metabolically susceptible target cells, as distinct from a direct action on immune cells alone. To test this hypothesis, we measured the impact of IP treatment on NKG2D ligand induction and assessed the functional effect of this ligand induction on NK cell cytotoxicity.

## Results and discussion

### IP induces NKG2D ligand expression

NKG2D ligand induction on target cells is required to trigger NKG2D signaling and NKG2D‐dependent cytotoxicity. We hypothesized that exposure of target cells to IP would result in increased target cell NKG2D ligand expression. We tested this hypothesis using the model of metabolic induction of NKG2D ligands that we have developed and validated 16. In this model, human embryonic kidney (HEK)‐293T cells, which are not cancer‐derived cells, cultured in medium with 5 mM glucose are metabolically activated by a step change in extracellular glucose concentration to 25 mM and this activation strongly upregulates MICA expression. Cells cultured in medium with 5 mM glucose demonstrated a dose‐dependent increase in the cell surface expression of the archetypal NKG2D ligand, MICA, with increasing IP concentration (Fig. 1B) and this increase was statistically significant (Fig. 1C). In addition to HEK293T cells, HT1080 cells (human fibrosarcoma), and HeLa cells (human cervical carcinoma) also showed dose‐dependent upregulation of cell surface MICA when cultured with IP (Fig. 1D–F).

To exclude the possibility that the change in cell surface MICA expression arose from a redistribution of intracellular MICA to the cell surface, we measured the effect of IP on cell surface MICA and on total cell MICA by flow cytometry of non‐permeabilized or fully permeabilized cells respectively. We found that IP led to increased MICA expression in both conditions, suggesting that the effect of IP on MICA is not due to a change in distribution of MICA between different cellular compartments (Fig. 1D). To control for cell permeabilization and to exclude the possibility of a nonspecific effect on global protein production, we measured proliferating cell nuclear antigen (PCNA) in non‐permeabilized and permeabilized cells by flow cytometry. PCNA was only detected in permeabilized cells, confirming that adequate permeabilization to the nucleus was achieved (Fig. 1E). PCNA levels did not increase with IP treatment, demonstrating that IP‐induced MICA expression is not a manifestation of a nonspecific effect on global protein production (Fig. 1E).

We measured the cell surface expression of the other NKG2D ligands MICB, and ULBP1‐5 and found substantial dose‐dependent IP induction of MICA, ULBP2, and ULBP3, with lesser changes seen for the ULBP1 and ULBP4 and no change seen for MICB or ULBP5 (Fig. 1F). This is similar to the pattern of NKG2DL expression we have previously reported in response to a shift to Warburg metabolism with increased purine nucleotide levels 16.

### IP induces cellular proliferation and synthetic activity

“Warburg” metabolism is characterized by increased glucose metabolism and increased biosynthetic activity in proliferating or metabolically active cells. We hypothesized that the effect of IP on NKG2D ligand expression is mediated by alterations of cellular metabolism and biosynthetic capacity.

**Figure 2:**
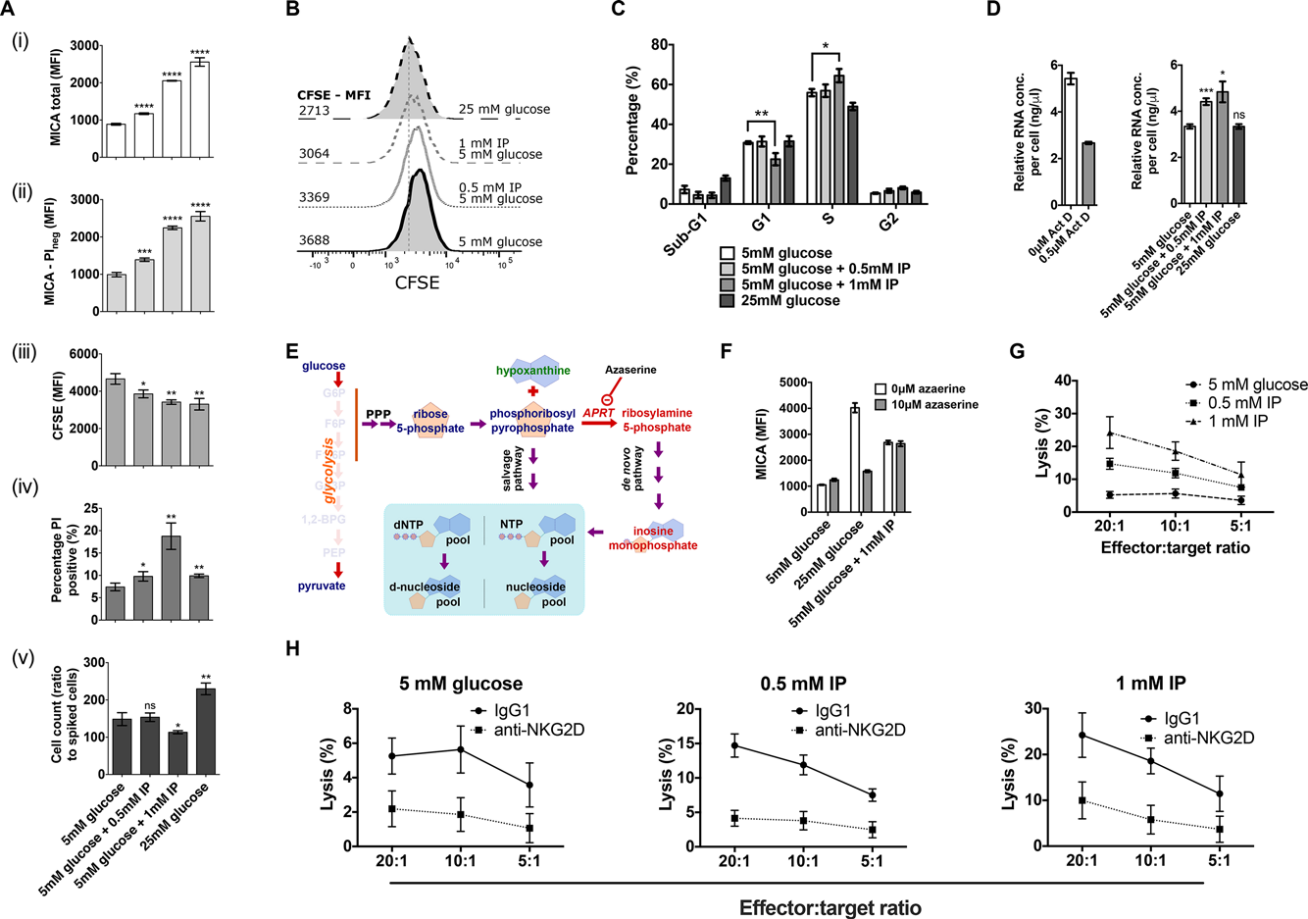
IP increases cell proliferation, alters cell cycle distribution, and enhances NKG2D‐dependent cellular cytotoxicity. (A) We measured the effect of IP on cell proliferation by flow cytometry. A significant dose‐dependent increase in cell surface expression was observed in the total cell population (i), and the intact PI‐negative population (ii). Mean CFSE fluorescence was significantly lower at the end of the culture period in cells treated with IP (iii). The percentage of PI‐positive cells also increased significantly with IP treatment (iv). The total number of cells, measured by flow cytometry, present at the end of the 48‐h culture period was significantly lower with 1mM IP (v). (B) A representative frequency distribution histogram of CFSE fluorescence for cells in A(iii). (C) PI‐based cell cycle analysis performed using flow cytometry demonstrates that in 1 mM IP, the percentage of cells in the G1 phase is significantly lower than in cells cultured in 5 mM glucose alone. This is matched by a similar increase in cells in the S phase. (D) We hypothesized that cells treated with IP may have increased cellular biosynthesis. Treatment of cells with the RNA synthesis inhibitor actinomycin D was used as a control. Cells cultured in IP had significantly higher mean RNA concentrations. (E) A cartoon showing the relationship between glucose metabolism and purine nucleotide synthesis. Azaserine inhibits amidophosphoribosyl transferase (APRT), which catalyzes a step that commits 5‐carbon sugars to the de novo purine nucleotide synthesis pathway. (F) We have previously shown that glucose induces MICA expression by supplying 5‐carbon sugars for purine nucleotide synthesis. While the de novo purine synthesis inhibitor azaserine prevented glucose‐induced MICA (2C10) expression, it failed to prevent IP‐induced MICA expression. (G) We tested the effect of IP on cellular immunogenicity using chromium‐release cytotoxicity assays. HEK293T cells were used as target cells. Cells cultured in IP were significantly more susceptible (*p* < 0.0001) to NK‐cell mediated cytotoxicity in a dose‐dependent manner across a range of effector:target ratios, as determined by ANOVA. Error bars represent the 95% confidence interval of eight biological replicates. (H) Anti‐NKG2D antibody (5528660) was used to block recognition through the NKG2D receptor on effector NK cells. An IgG1 isotype control was added to control NK cells. In each of the conditions shown, blocking with anti‐NKG2D lead to a significant reduction in killing (*p* < 0.0001) as determined by ANOVA. Error bars represent the 95% confidence interval of eight biological replicates. ^*^ *p* <0.05; ^**^ *p* < 0.01; ^***^ *p* <0.001; ^****^ *p* < 0.0001. Unless otherwise stated, histograms represent mean and 95% confidence interval. The data shown is from a single experiment that contained three biological replicates. Experiments were performed independently three times with consistent results. Means were compared using *t*‐tests. PPP, pentose phosphate pathway; APRT, amidophosphoribosyl transferase.

We first tested this by measuring proliferation in IP‐treated cells using a flow cytometry‐based CFSE proliferation assay (Fig. 2A). Unlike activated lymphocytes, HEK293T cells proliferate asynchronously and continuously, causing the population fluorescence intensity in CFSE‐stained cells to decrease over time at a rate linked to the rate of cellular proliferation. Cells treated with IP demonstrated dose‐dependent MICA induction as expected (Fig. 2A(i)). The upregulation of MICA expression was similar on PI‐negative membrane‐intact live cells (Fig. 2A(ii)). The rate of cell division, measured by the reduction in mean CFSE fluorescence, was significantly increased in cells treated with IP (Fig. 2A(iii)). A representative frequency distribution plot of CFSE fluorescence is shown in Fig. 2B. Cell death, assessed by the permeability of the cell membrane to PI was higher in cells treated with IP (Fig. 2A(iv)). At an intermediate IP concentration (0.5 mM), the increased rate of proliferation was balanced by an increase in cell death, resulting in no significant difference in net cell numbers at the end of the culture period. At high (1 mM) IP concentrations, the rate of cell death exceeded the increased proliferation rate, leading to a significant reduction in total viable cell numbers (Fig 2A(v)).

We next assessed the effect of IP on cell cycle distribution by flow cytometry (Fig. 2C). Cells cultured at a high IP concentration (1 mM) had a significantly lower proportion of cells in the G1 phase, and a corresponding increase in the proportion of cells in S phase, consistent with a shift toward increased biosynthesis in the cell population. This difference was reflected in an increase in cellular total RNA, with a dose‐dependent increase in RNA concentrations observed with IP treatment (Fig. 2D). Phosphoribosyl pyrophosphate, derived from early glycolytic intermediates through the pentose phosphate pathway, is committed to the de novo purine synthesis pathway by the enzyme amidophosphoribosyl transferase (APRT) (Fig. 2E). We previously demonstrated that glucose‐induced MICA expression is dependent on glucose‐induced purine synthesis, an effect that can be blocked by the APRT inhibitor azaserine 16. To test whether increased de novo purine synthesis was responsible for the impact of IP on MICA expression, we treated cells cultured with high glucose (25 mM) or IP with azaserine. While azaserine prevented glucose‐induced MICA expression, it failed to prevent IP‐induced MICA expression, suggesting that IP acts downstream or independently of de novo purine synthesis (Fig. 2F).

### IP increases NKG2D‐dependent cellular immunogenicity

We tested the effect of IP on cellular immunogenicity using an NK cell chromium release cytotoxicity assay. Cells cultured with IP had a significant dose‐dependent increase in NK‐mediated cytotoxicity across a range of effector:target ratios (*P* < 0.0001, Fig. 2G). We tested whether the increased IP‐induced immunogenicity was mediated through NKG2D recognition by blocking the NKG2D receptor on NK cells with an anti‐NKG2D antibody or an IgG1 isotype control. Incubation with anti‐NKG2D significantly reduced IP‐induced cytotoxicity (Fig. 2H), suggesting a significant proportion of IP‐induced cellular immunogenicity is NKG2D‐dependent.

### IP increases intracellular purine nucleotide and TCA cycle intermediate levels

**Figure 3:**
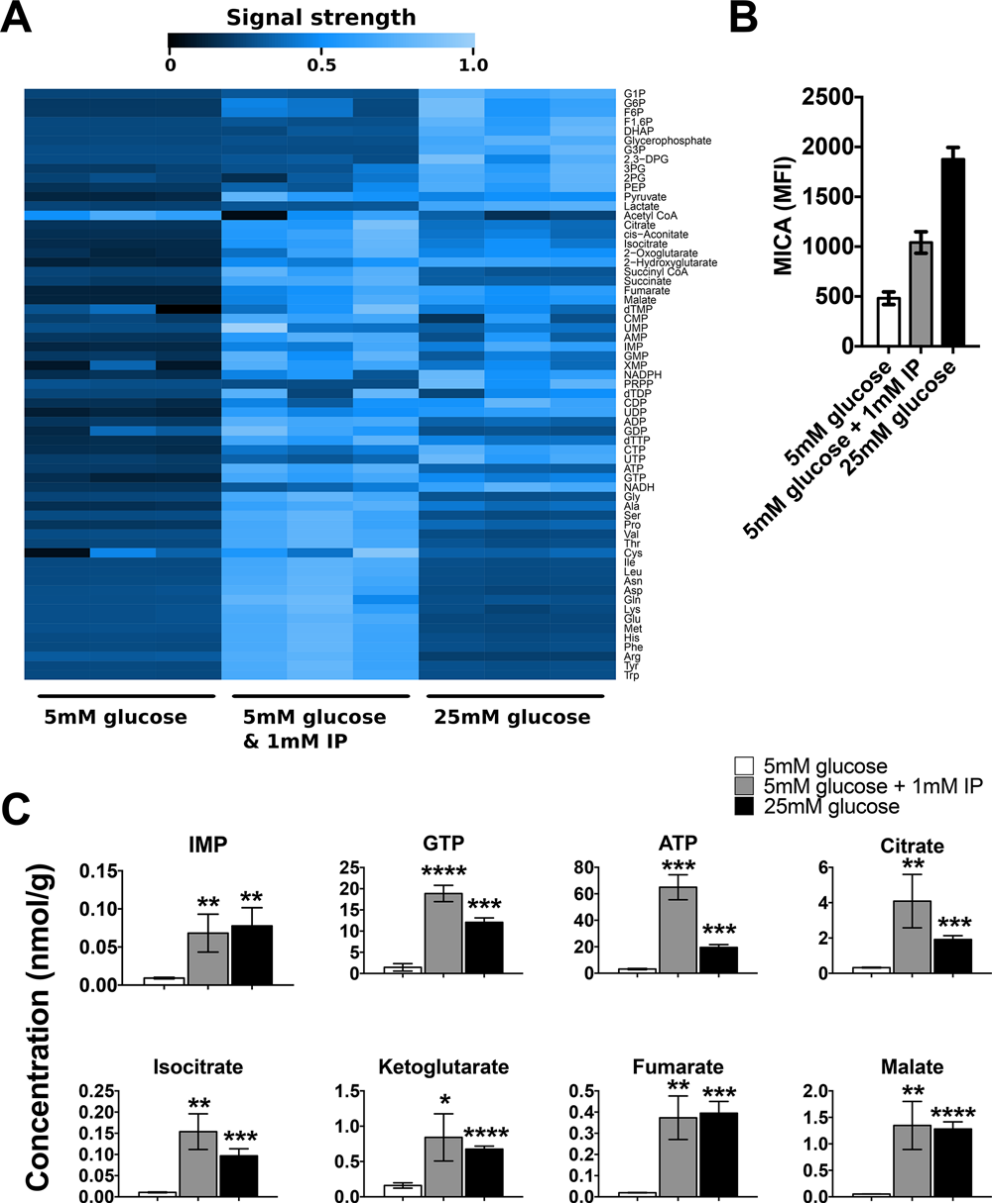
IP increases intracellular tricarboxylic acid (TCA) cycle and purine nucleotide concentrations. (A) Mass spectrometry‐based analysis of intracellular metabolite concentrations in IP‐treated cells demonstrated low concentrations of proximal glycolytic metabolites and high concentrations of TCA cycle metabolites, purine nucleotides and amino acids. (B) Cell surface MICA (2C10) expression, measured by flow cytometry, for samples analyzed in (A). (C) Analysis of metabolite concentrations depicted in (A) demonstrates significant IP‐induced increases in purine nucleotide and TCA cycle metabolite concentrations ^*^ *p* < 0.05, ^**^ *p* < 0.01, ^***^ *p* <0.001, ^****^ *p* < 0.0001. Histograms represent mean and 95% confidence interval of three biological replicates. One experiment was performed, as described in the methods section.

We have previously shown that purine nucleotide metabolism regulates MICA expression and that this regulation is associated with changes in the levels of intracellular purine metabolites and TCA cycle intermediates 16. Therefore, we undertook a metabolomic study of the effect of IP, using capillary electrophoresis time‐of‐flight mass spectrometry and liquid chromatography time‐of‐flight mass spectrometry to measure intracellular metabolite concentrations in cells treated with IP and this was compared to control data from cells cultured in 5 or 25 mM glucose (Fig. 3A). Cell surface MICA expression in these cells was assessed in parallel with the metabolomic profiling and confirmed IP‐ induced MICA expression (Fig. 3B). Culture of the cells in IP caused a significant increase in intracellular concentrations of purine nucleotide precursors (notably the common purine nucleotide precursor inosine monophosphate (IMP)), ATP, and GTP and the TCA cycle metabolites that are required to maintain purine nucleotides in their high‐phosphorylation state (Fig. 3C). These IP‐induced changes in intracellular metabolite concentrations are consistent with our previous observation that increased high energy purine nucleotide concentrations are sufficient and necessary to induce cell surface NKG2D ligand expression.

## Concluding remarks

We demonstrate that IP enhances NK killing through metabolic activation and induction of NKG2D ligand expression on the surface of target cells. IP is safe, is already licensed for human use, and may offer a straightforward approach to the upregulation of NKG2D ligands for potential therapeutic benefit. IP may serve as an adjunct to immunotherapy treatments. Further clinical studies could assess efficacy, dosage, and toxicities in this context.

## Materials and methods

### Cell culture

Unless otherwise stated, cells were grown in glucose free DMEM (Life Technologies, Paisley, UK) supplemented with 10% fetal calf serum, 2 mM pyruvate, penicillin/streptomycin, and glucose from a 2M stock solution at the concentration indicated. NK92 cells were cultured in RPMI supplemented with 10% fetal calf serum, 2 mM pyruvate, penicillin/streptomycin solution, and IL‐2 at a concentration of 200 units/mL.

### Chemicals and reagents

Glucose (#49152), azaserine (#11430), CFDA‐SE (#21888), PI (P4170), and actinomycin D (A1410) were purchased from Sigma–Aldrich (St Louis, MO). AICA‐Rs (#9944S) was purchased from New England Biolabs (Ipswich, MA). Chromium51 (NEZ030S001MC) was purchased from Perkin Elmer (Waltham, MA). Inosine pranobex was supplied by Newport Pharmaceuticals (Dublin, Ireland).

### Antibodies

MICA (2C10, IgG1), ULBP4 (6E6, IgG2B), and ULBP5 (6D10, IgM) were purchased from Santa Cruz (Santa Cruz, CA); MICB (MAB1599, IgG2b), ULBP1 (MAB1380, IgG2a), ULBP2 (MAB1298, IgG2a), and ULBP3 (MAB1517, IgG2a) were purchased from R&D Systems (Minneapolis, MN). Podoplanin (NZ‐1, IgG1) was purchased from AngioBio. HLA‐ABC (W6/32, IgG2a), Anti‐PCNA (14‐9910‐80, IgG2a), and isotype control antibodies were purchased from eBioscience (Hatfield, UK). Alexa fluor 647‐conjugated goat anti‐mouse IgM (A‐21238) and Alexa Fluor 647‐conjugated goat anti‐mouse IgM (A‐21236) were purchased from Invitrogen (Carlsbad, CA).

### Flow cytometry

Flow cytometry analysis was carried out on a BD FacsCanto flow cytometer (Franklin Lakes, NJ), and analyzed using FlowJo (Ashland, OR). A forward versus sidescatter window was used to draw a gate including the full population of cells. This gate was used to assess fluorescence in the target cell population (Fig. S1). Mean fluorescence intensity values were recorded.

Cell preparation and staining was carried out as previously described 16. Assessments of cell viability were made by adding PI to the final resuspension buffer at 4 µg/mL.

Total cell MICA was measured by permeabilized‐cell flow cytometry. Prior to primary staining, cells were fixed by resuspension in 2% paraformaldehyde for 30 min, and permeabilized with 0.05% saponin in PBSA for 30 min. The staining protocol was carried out as described above, but with 0.05% saponin added to each of the buffers.

### Cell cycle analysis

Cell cycle analysis was performed by resuspending cells in 1 mL of 1% paraformaldehyde (PFA) in PBSA and fixing on ice for 60 min. Cells were washed with ice‐cold PBSA and re‐suspended in PBSA. While vortexing, 4.5 mL of ice cold 70% ethanol was added. The samples were incubated at 4°C for 2 h and further washed in PBSA. Cell pellets were resuspended in PI staining solution containing 40 µg/mL PI, 100 µg/mL RNase A in PBS. The cells were incubated at 37°C for 30 min before flow cytometry. PI fluorescence was measured in linear mode. Doublets were excluded by initial PI width‐area gating. PI voltage was adjusted to center the G1 population on the 50kV area level. Cell cycle modeling was carried out using FlowJo.

### CFSE proliferation assay

For adherent cells, the culture medium was aspirated from each well, and replaced with a 5 µM CFDA‐SE solution. Cells were incubated at 37°C for 10 min, at which point the CFDA‐SE solution was aspirated and replaced with fresh culture medium as indicated. Cells were cultured for 48 h, before measuring CFSE fluorescence through a FITC filter by flow cytometry.

### Chromium release cytotoxicity assay

A total of 1 × 10^6^ target cells were washed in fresh culture medium, pelleted, and resuspended in  50 µL chromium‐51 (0.05 mCi) per sample and incubated for 1 h at 37°C. Labeled cells were washed twice with fresh RPMI and transferred to a 96‐well plate. NK92 cells were serially diluted to generate the effector:target ratios described. Background lysis was measured by adding 100µl of NK92 medium only, and maximal lysis was measured by adding 100 µL of 5% triton X. Effector and target cells were mixed by gentle pipetting, and co‐incubated at 37°C for 1 h. The reaction plate was centrifuged, and 25 µL of supernatant from each well was added to 150 µL of scintillation fluid in a fresh 96‐well plate. Scintillation counts were measured on a Microbeta TriLux liquid scintillation counter. To measure NKG2D specific killing, effector NK92 cells were initially resuspended in culture medium with 5 µg/mL anti‐NKG2D antibody (BD biosciences; 552866) for 30 min, before resuspension in RPMI, and testing as described above. An IgG1 isotype (eBioscience, 14‐4714‐85) was added to the control NK92 cells. Control samples at 5 and 25 mM glucose were prepared and analyzed in parallel and these datasets have been reported previously 16.

### Total RNA per cell measurement

Cells were cultured as indicated for 48 h. RNA was extracted from a 50% aliquot of each sample using the Trizol method and PureLink RNA mini kit (Life Technologies, Carlsbad, CA) with DNase digestion. RNA concentrations were measured using a Nanodrop 2000 (Wilmington, DE).

The second 50% aliquot was used to measure cell counts by flow cytometry. A separate population of cells was stained with 5µM CFDA‐SE and counted by hemocytometer. CFDA‐SE‐stained cells were used to pipette‐spike unstained test cell samples, immediately prior to flow cytometry. The number of test cells per 1000 CFDA‐SE positive cells was calculated. Measurements were made in biological triplicate. Cells treated with the RNA synthesis inhibitor, actinomycin D, was used as a control.

### Metabolomic analysis

The concentrations of intracellular anionic and cationic metabolites were measured by capillary electrophoresis time‐of‐flight mass spectrometry and 2‐oxoglutarate was measured by liquid chromatography time‐of‐flight mass spectrometry 22, 23. Samples were prepared as described previously 24. Control samples at 5 and 25 mM glucose for comparison were prepared and analyzed in parallel and these datasets have been reported previously 16. Results show independent biological triplicates, prepared in Oxford, UK, and analyzed in Keio, Japan.

### Statistical analysis

Histogram bars represent mean values and error bars represent the 95% confidence interval of the mean. Statistical significance and *p*‐values were calculated using t‐tests unless otherwise specified. Correlation between cell surface MICA expression and intracellular metabolite concentration was calculated using the correlation coefficient and coefficient of determination between mean values measured in parallel. Unless otherwise specified, ^∗^ *p* < 0.05, ^∗∗^ *p* < 0.01, and ^∗∗∗^ *p* < 0.001. Metabolomic analysis was undertaken using R 25.

## Conflict of Interest

The authors have no conflicting financial interests.

**Abbreviations**
CFDA‐SE: carboxyfluorescein diacetate succinimidyl ester
CFSE: carboxyfluorescein succinimidyl ester
FITC: fluorescein isothiocyanate
GTP: guanosine triphosphate
HEK: human embryonic kidney
IMP: inosine monophosphate
IP: inosine pranobex
LC‐TOFMS: liquid chromatography time‐of‐flight mass spectrometry
MICA: MHC class I polypeptide‐related sequence A
MICB: MHC class I polypeptide‐related sequence B
NKG2D: Natural killer group 2 D
PBSA: Phosphate‐buffered saline
PCNA: proliferating cell nuclear antigen
SSPE: Subacute sclerosing panencephalitis
TCA: tricarboxylic acid cycle
ULBP: UL16‐binding protein

## Supporting information

Figure S1 Gating strategy.
