# Supplementary material for: Inosine pranobex enhances human NK cell cytotoxicity by inducing metabolic activation and NKG2D ligand expression
Source: Eur J Immunol. 2019 Sep 12;50(1):130–7. doi: 10.1002/eji.201847948 (PMC6972573; doi:10.1002/eji.201847948)
Supplement: Supplementary file 1 — Figure S1 Gating strategy. [file EJI-50-130-s001.pdf]

European Journal of Immunology

Supporting Information
for
DOI 10.1002/eji.201847948

Michael T. McCarthy, Da Lin, Tomoyoshi Soga, Julie Adam and Christopher A. O'Callaghan

Inosine pranobex enhances human NK cell cytotoxicity by inducing metabolic activation and NKG2D ligand expression

© 2019 The Authors. *European Journal of Immunology* published by WILEY-VCH Verlag GmbH & Co. KGaA, Weinheim.

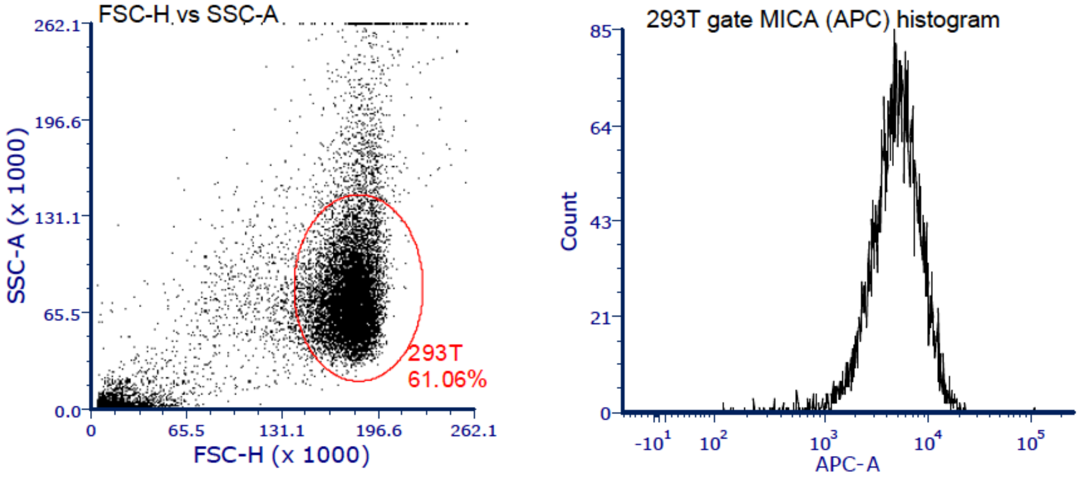

Figure S1 Gating strategy.
(A) An FSC-H vs SSC-A plot was created, and a gate to include the central 293T cell population was drawn. (B) A frequency distribution histogram was drawn using the 293T cell gate. The mean fluorescence intensity of the relevant marker was calculated from this frequency distribution.
